# Impact of victory and defeat on the perceived stress and autonomic regulation of professional eSports athletes

**DOI:** 10.3389/fpsyg.2022.987149

**Published:** 2022-08-25

**Authors:** Sergio Machado, Leandro de Oliveira Sant'Ana, Luis Cid, Diogo Teixeira, Filipe Rodrigues, Bruno Travassos, Diogo Monteiro

**Affiliations:** ^1^Department of Sports Methods and Techniques, Federal University of Santa Maria, Santa Maria, Brazil; ^2^Department of Sports Science, University of Beira Interior, Covilhã, Portugal; ^3^Laboratory of Physical Activity Neuroscience, Neurodiversity Institute, Queimados, Brazil; ^4^Postgraduate Program in Physical Education, Federal University of Juiz de Fora, Juiz de Fora, Brazil; ^5^Research Center in Sport, Health and Human Development (CIDESD), Vila Real, Portugal; ^6^Sport Sciences School of Rio Maior, Polytechnic of Santarém (ESDRM-IPSantarém), Rio Maior, Portugal; ^7^Life Quality Research Centre (CIEQV), Leiria, Portugal; ^8^Faculty of Physical Education and Sport, Lusófona University, Lisbon, Portugal; ^9^Research Center in Sport, Physical Education, and Exercise and Health (CIDEFES), Lisbon, Portugal; ^10^ESECS, Polytechnic of Leiria, Leiria, Portugal; ^11^Portugal Football School, Portuguese Football Federation, Cruz Quebrada, Portugal

**Keywords:** victory, defeat, eSports, perceived stress, heart rate variability, HRV

## Abstract

Competitive sports involve physiological, technical and psychological skills, which influence directly on individuals’ performance. This study aims to investigate the levels of perceived stress and Heart Rate Variability (HRV) before and after matches with victory and defeat in professional eSports athletes. Our hypothesis was that the winners would have better autonomic and stress responses after match, thus corroborating the literature on neurocardiac connections. Fifty male eSport players were selected players from 10 different Brazilian teams. The experiment was carried out in 2 sessions. Firstly, after signing the informed consent form, 24 h before the game, anthropometric, physical activity levels and time of expertise data were recorded only for sample characterization and the players were familiarized with the perceived stress scale—10 (PSS-10) and the HRV measurements. Secondly, players performed the PSS-10 and HRV recording at rest by 10 min 60 and 30 min before the game (i.e., baseline time) and 10 min after the end of the game. Overall, concerning PSS-10 our findings show that VG had significant reduced scores in post-game time compared to baseline (BL) and pre-game times, while DG had significant increased scores in post-game time compared to BL and pre-game times. Regarding HRV, our results demonstrate that VG had significant increase in RR, SDNN, rMSSD, pNN50 and HF, and significant decrease in LF and LF/HF, while DG had a significant decrease in RR, SDNN, rMSSD and HF, and significant increase in LF and LF/HF. It was observed that VG had better HRV responses (greater parasympathetic activation) as well as lower levels of perceived stress, while DG had worst HRV responses (greater sympathetic activation) and higher levels of perceived stress.

## Introduction

Independently of the environment and activities, individuals’ performance are always affected by their physiological (Knechtle, 2014; Oliveira-Silva et al., 2018), technical (Chidley et al., 2015) and psychological (Knechtle, 2014) skills. For example, as a psychological factor, stress management is one of the most reported factors that hinders performance, due to the impact on autonomic functions (Blásquez et al., 2009). Particularly in sport, studies have shown the negative influence of stress on athletes from different sports (Foster et al., 2001; Bara-filho et al., 2013) as well as, negatively affecting performance (Mamlouk et al., 2021). Within this context, a non-invasive and reliable method to assess autonomic control is through heart rate variability (HRV) in which are variations in the time interval between heartbeats (Salahuddin et al., 2018) are accessed. HRV has been used both in clinical and sports contexts, as it is low cost and easy to apply (Ogliari et al., 2015; Laborde et al., 2017).

With the analysis of HRV it is possible to examine the balance of autonomic control (i.e., between the sympathetic and parasympathetic nervous systems; Laborde et al., 2017), being considered an index of adaptive capacity. Since HRV increases (i.e., an indicator of good adaptation; Sant'Ana et al., 2020, 2022) stress tends to reduce. In line with that, HRV tends to decrease during moments of pre-competitive stress (Mamlouk et al., 2021), as well as before competitive matches (Goessl et al., 2017). Other studies (e.g., Hynynen et al., 2011), reveal that previous experience with stressful situations seems to minimize autonomic changes corroborating the importance of athletes’ previous training and competition history.

In addition, few studies have examined the psychophysiological behavior (e.g., Seong et al., 2004; Fuentes et al., 2018; McEwan et al., 2018), especially on the relationship between HRV and stress perception in athletes (Oliveira-Silva et al., 2018; Mamlouk et al., 2021). However, despite being important variables to compose a sports performance evaluation (Broodryk et al., 2021), further research is still needed to expand information due to the close relationship between autonomic and emotional behaviors (Kemp and Quintana, 2013) both for performance evaluation, and recovery between games and throughout the competition. Stress, like other types of emotional reactions, is associated with a non-specific reaction of the body to any type of demand (Barnes and Van Dyne, 2009) and depending on the level of stress, will affect cardiac functions, mediated by neurological pathways (Friedman, 2007) that can acutely and/or chronically modify the autonomic condition (Goessl et al., 2017). In sports, athletes suffer strong mental demands, and the result of a competition can be a reason for significant changes in psychophysiological functioning (Broodryk et al., 2021).

As eSports athletes compete in environments of high pressure and competitiveness, which are very similar to more traditional sports environments (Machado et al., 2021), they need to develop their mental skills, as well as the techniques used in the game to achieve optimal performance. Furthermore, they need strategic thinking, motivation, quick decision-making and intelligence, sustained attention, planning, working memory and inhibitory control, adapt to their opponents, communicate properly with teammates and trust your abilities. All these factors contribute to or affect the psychological state of players (Himmelstein et al., 2017).

Despite of that, the literature on eSports is still scarce, with studies based mainly on reports about playing video games, but not about eSports, and associating eSports with unhealthy lifestyles and health-related problems. Therefore, there are several gaps in the literature to be explored, on cardiovascular, respiratory, metabolic (Monteiro Pereira et al., 2022) and psychological health (Trotter et al., 2021). In line with this, although there are some studies on autonomic functioning, more specifically HRV, and stress in the context of more traditional sports (Oliveira-Silva et al., 2018; Broodryk et al., 2021; Mamlouk et al., 2021), there is no published study in the literature on stress and HRV professional eSports athletes and particularly in victory and defeat.

Thus, this study aims to investigate the levels of perceived stress and HRV before and after matches with victory and defeat in professional eSports athletes. Our hypothesis was that the winners would have better autonomic and stress responses after match, thus corroborating the literature on neurocardiac connections (Reigal et al., 2018; Broodryk et al., 2021; Mamlouk et al., 2021). The study reported here serves as a basis for future research and to fill the current gap in knowledge, helping to understand the behavior of HRV and stress in eSports athletes in terms of victory and defeat. For the sake of knowledge, this is the first study to assess the effect of win and loss on both the autonomic and perceived functioning of professional eSports athletes.

## Materials and methods

### Participants

The sample size was determined through power calculations conducted with G*Power v.3.1 (Faul et al., 2009), using the following input parameters: medium anticipated effect size for a comparison between two dependent means (*d* = 0.50), statistical power 1−*β* = 0.80, and *α* = 0.05. Given that no study has presented results directly relevant to the effect being targeted in the present study, estimates of effect sizes pertaining to psychophysiological responses to similar experimental manipulations within the context of pre-competitive stress in sports science were used (i.e., Oliveira-Silva et al., 2018). Therefore, prior effect sizes in the reported study were found to be large (Oliveira-Silva et al., 2018). Based on these calculations, the target sample size determined for present study was of 20 (1−*β* = 1.616).

Fifty male eSport players aged 18–29 years (age: 24.98 ± 2.59 y, height: 178.6 ± 1.45 cm, weight = 78.5 ± 2.35 kg, time of experience: 7.68 ± 1.33 years, and physically inactive: 36 ± 1.05 min per week) of Brazilian teams took part of the study. The inclusion criteria were being professional athlete with at least 5 years of experience of national or international competitions, and 8–10 h of training daily. The exclusion criteria were to have neuropsychiatric, cardiovascular, or osteoarticular diseases, used any kind of neuropsychiatric drugs, used any caffeinated or alcoholic beverages on the day of the experiment. The study was approved by the Ethics Committee of the Research Center in Sport, Health and Human Development (CIDESD; Portugal), and all athletes were informed of the inherent risks and benefits of the study before signing an informed consent form.

### Experimental design

We selected players from 10 different Brazilian teams, all composed of 5 players. If any of the players did not meet the inclusion criteria, the team was not selected to participate in the study. We chose to always carry out the study with two teams per decisive game to facilitate the equalization of the sample to observe the phenomenon of victory and defeat. Thus, after the end of the experiment we had 25 players to Victory Group (VG) and Defeat Group (DG).

**Figure 1:**
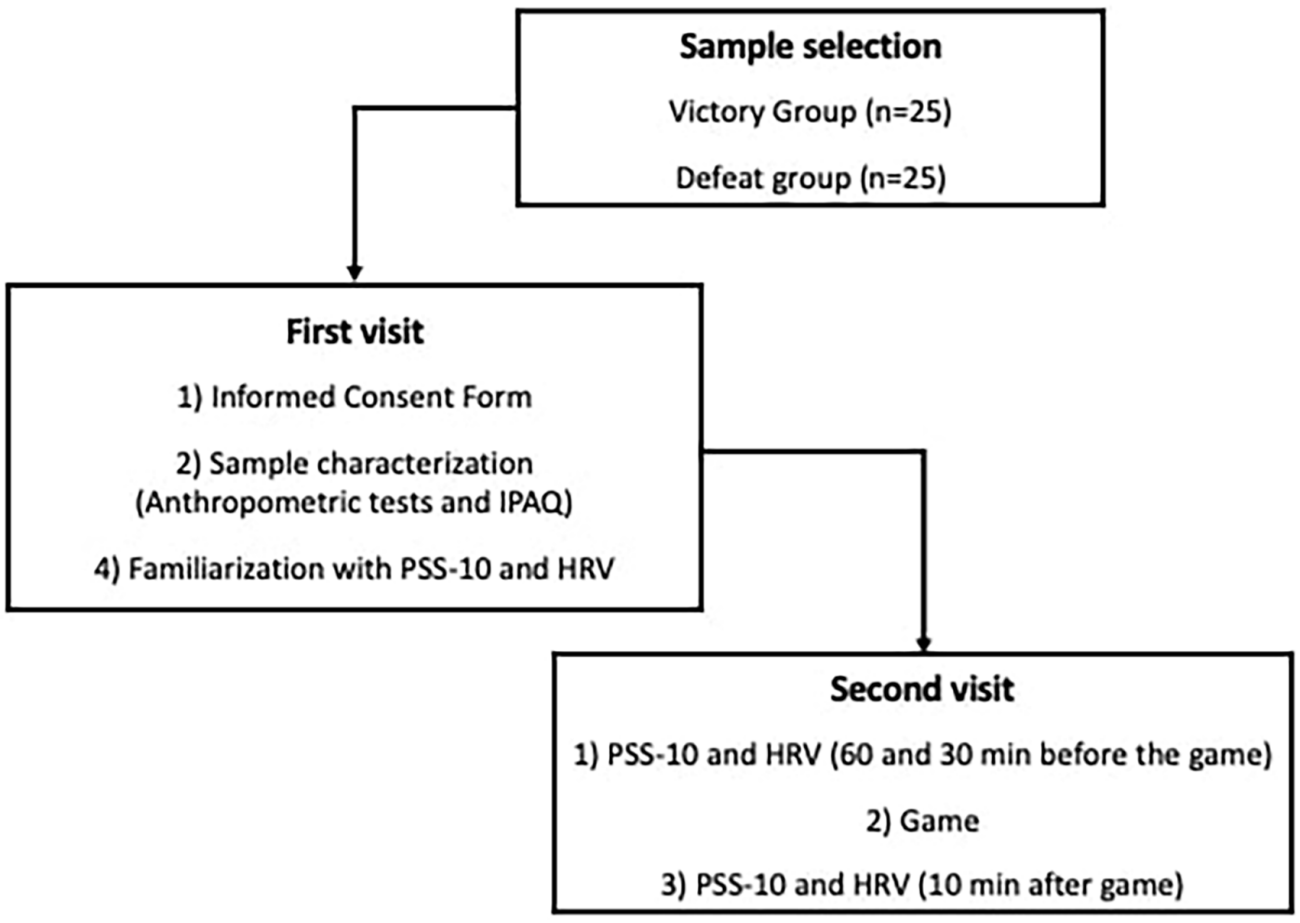
Experimental design.

Players were exposed to decisive games in international championships, such as CS: GO PGL Major Championship Fall and Six Invitational. The experiment was carried out in 2 sessions. In the first one, after signing the informed consent form in the game house, 24 h before the game, anthropometric (i.e., age, height and weight), physical activity levels through International Physical Activity Questionnaire (IPAQ; Hallal and Victora, 2004) and time of expertise data were recorded only for sample characterization and the players were familiarized with the perceived stress scale – 10 (PSS-10) and the HRV measurements. In the second one, the players performed the PSS-10 (Reis et al., 2010) and HRV recording at rest by 10 min, 60 and 30 min before the game (i.e., baseline [BL] and pre-game times, respectively) and 10 min after the end of the game (post-game time, Figure 1). Both sessions were carried out in the game house between 14:00–17:00 h to avoid circadian effects on psychological and autonomic performance. Psychological variables and HRV were assessed by the same researcher.

### Familiarization

The familiarization procedures with the PSS-10 occurred with the same researcher, 24 h before the experimental conditions, as follows: (1) the researcher read the specific instructions for all questions; (2) the researcher clarified that “there is no right or wrong answer” to the questions and that the answers must be given among the alternatives exposed; (3) the researcher determined for players that there could be no double answers to the same question, and highlighted the importance of veracity in the answers; (4) the researcher asked the players to review their answers before completing the questionnaire. This procedure occurred only during the familiarization phase. In turn, during the experimental conditions the questionnaire was self-reported.

As for the HRV collections, the players received instructions on the placement of the chest strap (over the xiphoid process), use of the heart rate monitor and received instructions to remain quiet, with eyes opened, and to breathe spontaneously over the acquisition period.

### Perceived stress scale-10

To assess perceived stress, the players responded to the PSS-10. We chose the PSS-10, which is a brief, easy-to-use version with equivalent psychometric properties to the PSS-14, as advised by Cohen and Williamson (1988). PSS-10 consists of 10 questions to verify how unpredictable, uncontrollable and overloaded participants perceive their lives. These three factors have been considered as central components in stress experience. PSS-10 is a general scale, which can be used in several age groups, from adolescents to elderly, as it does not contain specific issues of the context. The absence of specific context issues is an important factor in the scale and probably the reason this scale has been validated in several cultures, as well as in Brazilian culture (Reis et al., 2010). Each question presents 4 alternatives of response by Likert Scale of 1 (never) to 4 (always). The questions with positive response (4, 5, 6, 7, 9, 10 and 13) have their reverse score, as follows, 0 = 4, 1 = 3, 2 = 2, 3 = 1 and 4 = 0. The questions with negative response (1, 2, 3, 8, 11 and 12) should be added directly. The total scale is the sum of the scores of the 10 questions and the scores may range from 0 to 40 (scores of 0—13 is considered as low perceived stress, 14—26 as moderate perceived stress, and 27—40 as high perceived stress). The Brazilian version of PSS-10 showed good psychometric properties for perceived stress in Brazilian adults (Reis et al., 2010). The duration of the assessment was up to 10 min. The Cronbach’s α in our sample was 0.82.

### HRV recording and analysis

The HRV data from all players were recorded with players seated on a bench, in an air-conditioned room (Holmes et al., 2020). After assessment of perceived stress (≈5 min), players received the moistened strap transmitters and fitted firmly to the chest. Subsequently, players checked the functioning of the heart rate monitor receiver for RR intervals acquisition (Geus et al., 2019).

HRV was recorded at rest using a cardiotachometer Polar RS800cx (Polar™, Kempele, Finland) at a sampling of 1,000 Hz (Quintana et al., 2012b). A period of 10-min for the recording of HRV was performed (i.e., 5-min stabilization period and a 5-min post-stabilization period). Data corresponding to 5-min post-stabilization were extracted and downloaded for analysis by specific software (Polar Precision Performance, Polar™, Kempele, Finland). HRV indices were analyzed using the Kubios™ HRV software (Biomedical Signal Analysis Group, Department of Applied Physics, University of Kuopio, Kuopio, Finland) considering the 5-min post-stabilization (Draghici and Taylor, 2016). Data were visually inspected to identify artifacts (≤2%), which were manually removed with the interpolated adjacent RR interval values (filter power < medium; Johnston et al., 2020).

The dependent variables were analyzed in the domain of frequency (Low Frequency [LF], High Frequency [HF], and simpato-vagal balance [LF/HF]) and time (beat-to-beat intervals [R-R], standard deviation of the mean of qualified NN-interval [SDNN]. Proportion of successive NN intervals with a difference greater than 50 ms [pNN50], and root-mean-square difference of successive normal RR intervals [rMSSD] were also measured (Johnston et al., 2020).

### Statistical analyses

Assumptions of the homogeneity of variance and residual normality were tested by using the Levene’s and Shapiro–Wilk tests, respectively. Data with residual normality were represented by mean and standard deviation (M ± SD). At BL, the assumptions were met for age, weight, height, time of experience, PSS-10, and HRV. Thus, the independent samples t-tests were used to verify the differences between the two groups (VG vs. DG) in BL. A 2 × 2 mixed factor analysis of variance (ANOVA) was used to test for differences between Victory Group vs. Defeat Group (between-group effects) and differences among BL, pre-game, and post-game (within-group effects) for PSS-10, and HRV measurements in the time and frequency domains. Post-hoc analysis was performed using the Bonferroni to assess the effects within each group. The level of significance was determined by 5% (*p* < 0.05). The effect size was calculated and then interpreted as suggested by Cohen’s d—0.00 to 0.19 (trivial); 0.20 to 0.49 (small); 0.50 to 0.79 (moderate); and ≥ 0.80 (large; Cohen and Williamson, 1988). For correlation analysis between PSS-10 and HRV measures we used a bivariate Pearson’s correlation (Schober et al., 2018). All data were statistically treated by GraphPad Prism software, version 8.0.1.

## Results

### Sample characteristics

The groups in analysis revealed homogeneity. There were no significant group differences considering age, weight, height and time of experience. Descriptive data and differences between groups are shown in Table 1.

**Table 1:** Sample characteristics for VG and DG.

Variables	Victory group (*n* = 25)	Defeat group (*n* = 25)	Statistical difference
*Physical activity level*	M ± SD	M ± SD	*p*
Min per week	37 ± 1.2	35 ± 0.9	0.63
*Anthropometry*	M ± SD	M ± SD	*p*
Age (y)	25.04 ± 2.77	24.92 ± 2.41	0.87
Weight (kg)	79.6 ± 2.1	77.4 ± 2.6	0.78
Height (cm)	179.5 ± 1.6	177.7 ± 1.3	0.69
*Time of expertise*	M ± SD	M ± SD	*p*
Years (y)	7.92 ± 1.29	7.44 ± 1.38	0.22

y, years; kg, kilograms; cm, centimeters; M, mean; SD, standard deviation; *p*, *p*-value.

### PSS-10

**Figure 2:**
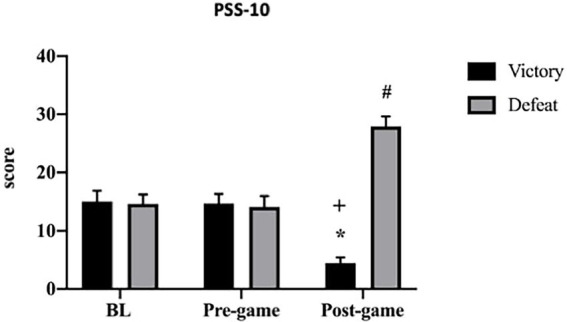
PSS-10 representation for victory and defeat groups. ^*^Significant difference compared to BL and Pre-game times (*p* ≤ 0.0001), ^+^Significant difference compared to Post-game time (*p* ≤ 0.0001), ^#^Significant difference compared to BL and Pre-game times (*p* ≤ 0.0001).

Any difference was found between groups in the BL (*p* = 0.823) and pre-game (*p* = 0.541) times, nor intra group difference between BL and pre-game times for VG (*p* = 0.999) and for DG (*p* = 0.989). PSS-10 in the post-game time was lower in VG than in DG (*p* ≤ 0.001; *d* = 16.62, CI 95%: 13.31 to 18.92, Figure 2).

Mixed analysis of variance showed significant group by time interaction [*F*(2, 144) = 889.8; *p* ≤ 0.001], main effects for group [*F*(1, 144) = 790.1; *p* ≤ 0.01], and time [*F*(2, 144) = 15.36; *p* ≤ 0.001] for PSS-10. The interaction revealed a decreased score for PSS-10 in the post-game (4.33 ± 0.96) compared to BL (15.12 ± 1.77) and pre-game (14.75 ± 1.62) times for VG (*p* ≤ 0.001; *d* = 7.58, CI 95%: 5.90 to 9.02 and *d* = 7.83, CI 95%: 6.09 to 9.31 respectively), while also showed an increased score for PSS-10 in the post-game (27.79 ± 1.71) compared to BL (14.70 ± 1.60) and pre-game (14.20 ± 1.84) times for DG (*p* ≤ 0.001; *d* = 7.91, CI 95%: 6.16 to 9.40 and *d* = 7.91, CI 95%: 5.95 to 9.11, respectively).

### Heart rate variability

**Figure 3:**
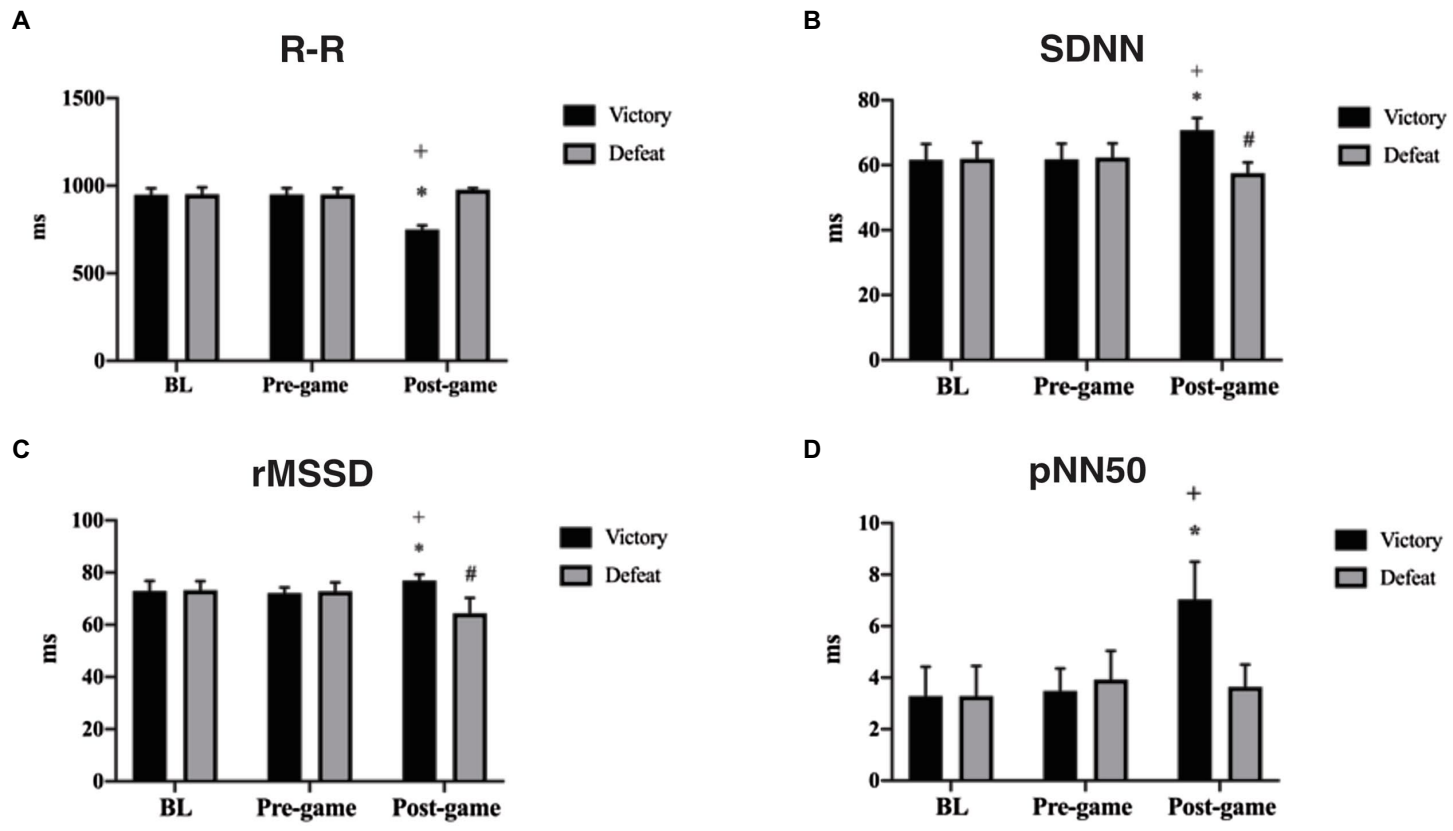
R-R, SDNN, rMSSD and pNN50 representations for victory and defeat groups. **(A)** R-R interval: ^*^Significant difference compared to BL and Pre-game times (*p* ≤ 0.0001), ^**+**^Significant difference compared to Post-game time (*p* ≤ 0.0001); **(B)** SDNN: ^*^Significant difference compared to BL and Pre-game times (*p* ≤ 0.0001)., ^**+**^Significant difference compared to Post-game time (*p* ≤ 0.0001), ^#^Significant difference compared to BL and Pre-game times (*p* = 0.001 and *p* ≤ 0.0001, respectively); **(C)** rMSSD: ^*^Significant difference compared to BL and Pre-game times (*p* ≤ 0.0001; *p* = 0.003 respectively), ^**+**^Significant difference compared to Post-game time (*p* ≤ 0.0001), ^#^Significant difference compared to BL and Pre-game times (*p* ≤ 0.0001); **(D)** pNN50: ^*^Significant difference compared to BL and Pre-game times (*p* ≤ 0.0001), ^**+**^Significant difference compared to Post-game time (*p* ≤ 0.0001).

Concerning time domain measures, for R-R any difference was found between groups in the BL (*p* = 0.994) and pre-game (*p* = 0.996) times, nor intra group difference between BL and pre-game times for VG (*p* = 0.999) and for DG (*p* = 0.999). In opposition, R-R in the post-game time was higher in VG than in DG (*p* ≤ 0.001; *d* = 15.02, CI 95%: 11.83 to 17.73, Figure 3A). Mixed analysis of variance showed for R-R interval significant group by time interaction [*F*(2, 144) = 226.9], main effects for group [*F* (1, 144) = 237.7], and main effects for time [*F* (2, 144) = 95.08]. The interaction revealed an increased score of R-R in the post-game (993.44 ± 4.63) compared to BL (950.4± 39.97) and pre-game (951.28 ± 36.15) times for VG (*p* ≤ 0.0001; *d* = 1.51, CI 95%: 0.86 to 2.12 and (*p* ≤ 0.0002; *d* = 1.64, CI 95%: 0.97 to 2.25 respectively), while also showed a decrease in the score of RR in the post-game (749.96 ± 22.46) compared to BL (948.36 ± 37.02) and pre-game (949.52 ± 33.56) times for DG (*p* ≤ 0.001; *d* = 6.48, CI 95%: 5.01 to 7.75 and *d* = 6.99, CI 95%: 5.42 to 8.34, respectively).

For SDNN, no difference was found between groups in the BL (*p* = 0.991) and pre-game (*p* = 0.966) times, nor intra group difference between BL and pre-game times for VG (*p* = 0.999) and for DG (*p* = 0.999). However, SDNN in the post-game time was higher in VG than in DG (*p* ≤ 0.001; *d* = 3.73, CI 95%: 2.76 to 4.58, Figure 3B). Mixed analysis of variance showed for SDNN significant group by time interaction [*F*(2, 144) = 39.82; *p* ≤ 0.001], main effects for group [*F*(1, 144) = 32.83; *p* ≤ 0.001], and main effects for time [*F*(2, 144) = 4,263; *p* = 0.001]. The interaction revealed an increased score of SDNN in the post-game (70.76 ± 3.75) compared to BL (61.6 ± 4.91) and pre-game (61.76 ± 4.9) times for VG (*p* ≤ 0.001; *d* = 2.13, CI 95%: 1.41 to 2.79 and *d* = 2.06, CI 95%: 1.35 to 2.71, respectively), while also showed a decreased score of SDNN in the post-game (57.48 ± 3.36) compared to BL (61.92 ± 4.97) and pre-game (62.28 ± 4.41) times for DG (*p* = 0.001; *d* = 1.05, CI 95%: 0.44 to 1.62 and *p* ≤ 0.001; *d* = 1.22, CI 95%: 0.61 to 1.81, respectively).

For rMSSD, no difference was found between groups in the BL (*p* = 0.990) and pre-game (*p* = 0.899) times, nor intra group difference between BL and pre-game times for VG (*p* = 0.999) and for DG (*p* = 0.999). However, rMSSD in the post-game time was higher in VG than in DG (*p* ≤ 0.001; *d* = 2.79, CI 95%: 1.97 to 3.52, Figure 3C). Mixed analysis of variance showed for rMSSD significant group by time interaction [*F* (2, 144) = 50.92; *p* ≤ 0.001], main effects for group [*F*(1, 144) = 40.26; *p* ≤ 0.001], and main effects for time [*F*(2, 144) = 5,646; *p* ≤ 0.001]. The interaction revealed an increased score of rMSSD in the post-game (76.96 ± 2.31) compared to BL (72.96 ± 3.80) and pre-game (72.12 ± 2.18) times for VG (*p* ≤ 0.001; *d* = 1.27, CI 95%: 0.65 to 1.86 and *p* = 0.003; *d* = 2.15, CI 95%: 1.43 to 2.81, respectively), while also showed a decreased score of rMSSD in the post-game (64.4 ± 5.93) compared to BL (73.24 ± 3.46) and pre-game (72.8 ± 3.42) times for DG (*p* ≤ 0.001; *d* = 1.82, CI 95%: 1.14 to 2.45 and *d* = 1.74, CI 95%: 1.06 to 2.36, respectively).

For pNN50, no difference was found between groups in the BL (*p* = 0.999) and pre-game (*p* = 0.422) times, nor intra group difference between BL and pre-game times for VG (*p* = 0.999) and for DG (*p* = 0.510). In opposition, pNN50 in the post-game time was higher in VG than in DG (*p* ≤ 0.001; *d* = 2.85, CI 95%: 2.03 to 3.95, Figure 3D). Mixed analysis of variance showed for pNN50 significant group by time interaction [*F*(2, 144) = 43.95; *p* ≤ 0.001], main effects for group [*F*(1, 144) = 29.06; *p* ≤ 0.0001], and main effects for time [*F*(2, 144) = 47.17; *p* ≤ 0.001]. The interaction revealed an increased score of pNN50 in the post-game (7.04 ± 1.45) compared to BL (3.28 ± 1.13) and pre-game (3.48 ± 0.87) times for VG (*p* ≤ 0.001; *d* = 2.89, CI 95%: 2.06 to 3.83 and *d* = 2.98, CI 95%: 2.13 to 3.73 respectively), while also showed no significant changes in the score of pNN50 in the post-game (3.64 ± 0.86) compared to BL (3.38 ± 1.18) and pre-game (3.92 ± 1.11) times for DG (*p* = 0.988 and *p* = 0.999, respectively).

**Figure 4:**
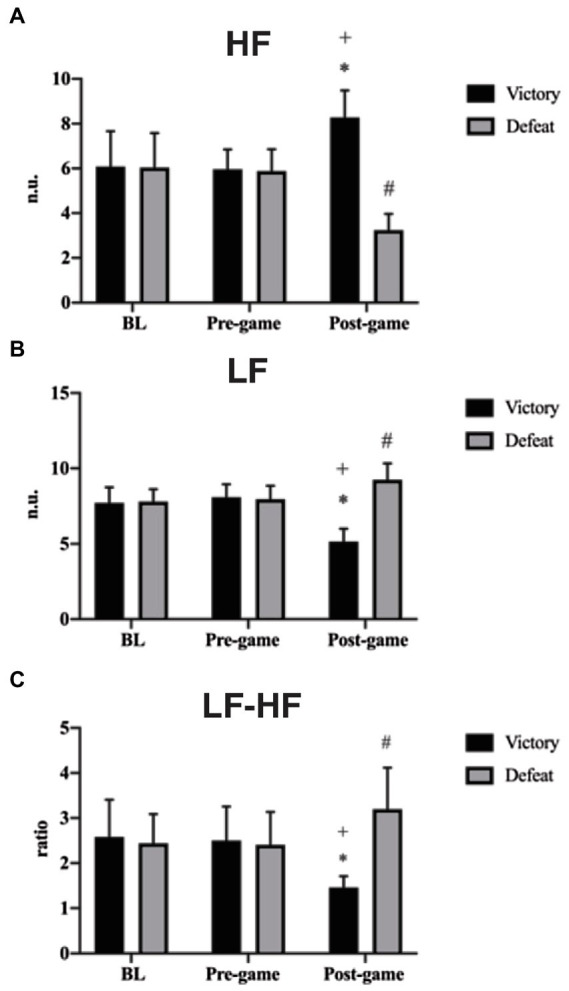
HF, LF and LF-HF representations for victory and defeat groups. **(A)** HF: ^*^Significant difference compared to BL and Pre-game times (*p* ≤ 0.0001), ^+^Significant difference compared to Post-game time (*p* ≤ 0.0001), ^#^Significant difference compared to BL and Pre-game times (*p* ≤ 0.0001); **(B)** LF: ^*^Significant difference compared to BL and Pre-game times (*p* ≤ 0.0001), ^+^Significant difference compared to Post-game time (*p* ≤ 0.0001), ^#^Significant difference compared to BL and Pre-game times (*p* ≤ 0.0001); **(C)** LF-HF: ^*^Significant difference compared to BL and Pre-game times (*p* ≤ 0.0001), ^+^Significant difference compared to Post-game time (*p* ≤ 0.0001), ^#^Significant difference compared to BL and Pre-game times (*p* = 0.004 and *p* = 0.002 respectively).

Considering frequency domain measures, for HF any difference was found between groups in the BL (*p* = 0.993) and pre-game (*p* = 0.999) times, nor intra group difference between BL and pre-game times for VG (*p* = 0.999) and for DG (*p* = 0.999). HF in the post-game times was higher in VG than in DG (*p* ≤ 0.0001; *d* = 5.09, CI 95%: 3.89 to 6.14, Figure 4A). Mixed analysis of variance showed for HF significant group by time interaction [*F*(2, 144) = 72.24; *p* ≤ 0.001], main effects for group [*F*(1, 144) = 77.55; *p* ≤ 0. 001], and main effects for time [*F*(2, 144) = 10.22; *p* ≤ 0.001]. The interaction revealed an increased score of HF in the post-game (8.28 ± 1.20) compared to BL (6.08 ± 1.57) and pre-game (5.96 ± 0.88) times for VG (*p* ≤ 0.001; *d* = 1.57, CI 95%: 0.92 to 2.18 and *d* = 2.20, CI 95%: 1.47 to 2.87, respectively), however with no significant differences between times for while also showed a decreased score of HF in the post-game (3.24 ± 0.72) compared to BL (6.04 ± 1.54) and pre-game (5.88 ± 0.97) times for DG (*p* ≤ 0.001; *d* = 2.33, CI 95%: 1.58 to 3.01 and *d* = 3.09, CI 95%: 2.23 to 3.86, respectively).

For LF, no difference was found between groups in the BL (*p* = 0.986) and pre-game (*p* = 0.956) times, nor intra group difference between BL and pre-game times for VG (*p* = 0.941) and for DG (*p* = 0.999). However, LF in the post-game time was lower in VG than in DG (*p* ≤ 0.0001; *d* = 4.15, CI 95%: 3.13 to 5.09, Figure 4B). Mixed analysis of variance showed for LF significant group by time interaction [*F*(2, 144) = 81.64; *p* ≤ 0.001], main effects for group [*F*(1, 144) = 79.13; *p* ≤ 0.001] and main effects for time [*F*(2, 144) = 10.22; *p* ≤ 0.001]. The interaction revealed a decreased score of LF in the post-game (5.16 ± 0.85) compared to BL (7.72 ± 1.02) and pre-game (8.08 ± 0.86) times for VG (*p* ≤ 0.001; *d* = 2.73, CI 95%: 1.92 to 3.45 and *d* = 3.42, CI 95%: 2.50 to 4.22, respectively), while also showed an increased score of LF in the post-game (9.24 ± 1.09) compared to BL (7.8 ± 0.81) and pre-game (7.96 ± 0.88) times for DG (*p* ≤ 0.001; *d* = 1.50, CI 95%: 0.85 to 2.10 and *d* = 1.29, CI 95%: 0.66 to 1.88, respectively).

For LF-HF, no difference was found between groups in the BL (*p* = 0.870) and pre-game (*p* = 0.952) times, nor intra group difference between BL and pre-game times for VG (*p* = 0.999) and for DG (*p* = 0.999). In opposition, LF-HF in the post-game time was lower in VG than in DG (*p* ≤ 0.001; *d* = 2.59, CI 95%: 1.80 to 3.30, Figure 4C). Mixed analysis of variance showed for LF-HF significant group by time interaction for [*F*(2, 144) = 27.55; *p* ≤ 0.001], and main effects for group [*F*(1, 144) = 18.02; *p* ≤ 0.001]. The interaction revealed a decreased score of LF-HF in the post-game (1.42 ± 0.20) compared to BL (2.52 ± 0.78) and pre-game (2.44 ± 0.70) times for VG (*p* ≤ 0.001; *d* = 1.93, CI 95%: 1.23 to 2.57 and *d* = 1.98, CI 95%: 1.28 to 2.62, respectively), while also showed an increased score of LF-HF in the post-game (3.18 ± 0.94) compared to BL (2.41 ± 0.65) and pre-game (2.34 ± 0.66) times for DG (*p* = 0.004; *d* = 0.95, CI 95%: 0.35 to 1.52 and *p* = 0.002; *d* = 1.03, CI 95%: 0.43 to 1.61, respectively).

Concerning correlation analyses among PSS-10 and HRV indexes, we did not find any significative difference.

## Discussion

The aim of present study was to verify the levels of perceived stress and HRV before and after decisive games involving victory and defeat in professional eSports athletes. In line with our hypothesis, VG had better autonomic and stress responses in comparison to DG that had worst autonomic and stress responses after game. Although there is evidence of psychophysiological aspects in athletes (Follador et al., 2018), there is still a need for more studies investigating HRV and stress behavior in athletes, especially in eSports athletes, who have strong psychological demands (Machado et al., 2021).

Recent studies have highlighted the importance of analyzing HRV and the state of stress in athletes (Britton et al., 2019) and also how these variables are in constant association (Moreira et al., 2021). In this regard, the present study showed that winning or losing can interfere with autonomic and stress responses. Overall, concerning PSS-10 our findings show that VG had significant reduced scores in post-game time compared to BL and pre-game times, while DG had significant increased scores in post-game time compared to BL and pre-game times. In addition, there were no differences in BL and pre-game times, as well as between BL and pre-game for both VG and DF in PSS-10. Regarding HRV, our results demonstrate that VG had significant increase in RR, SDNN, rMSSD, pNN50 and HF, and significant decrease in LF and LF/HF, while DG had a significant decrease in RR, SDNN, rMSSD and HF, and significant increase in LF and LF/HF. In addition, there were no differences in BL and pre-game times, as well as between BL and pre-game for both VG and DF in any measure.

The findings of the present study corroborate what the literature presents about HRV and stress behavior, where these variables are reactive to different emotions (Cooke et al., 2011), and that depending on the situation, they can offer relevant response magnitudes due to a strong association between the autonomic nervous system and psychological state, well described in the literature (Kemp and Quintana, 2013). Within this context, the outcome of a competition can significantly contribute to physiological and psychological responses (Salvador et al., 2003). Studies have highlighted the importance of investigating psychophysiological variables in athletes (Britton et al., 2019) and HRV has been relevant in monitoring athletes (Nakamura et al., 2015). Likewise, perceived stress in high-performance sports is extremely important for emotional control throughout the competition (Oliveira-Silva et al., 2018). However, HRV can offer changes due to emotional behavior, mainly due to stress (Goessl et al., 2017) and thus, we can highlight that these psychophysiological behaviors affect the athlete’s performance (Alfonso and Capdevila, 2022). Considering information about victory and defeat, there are few studies for this discussion (Broodryk et al., 2021). However, we can suggest that different outcomes, such as victory and defeat, can affect positively and negatively on physiological (HRV) and psychological (stress) behaviors.

Therefore, our findings are in line with previous studies (e.g., Broodryk et al., 2021) who found that soccer players showed positive responses to victory and negative responses to defeat, in physiological (hormonal behavior) and psychological (anxiety and mood) variables. That is, the eSports winner players tend to reveal more adjusted responses in the perception of stress and greater parasympathetic activation compared to losers.

Regarding possible mechanisms underlying perceived stress, sympathetic/parasympathetic imbalance is related to performance of tasks (Barnes and Van Dyne, 2009), and depending on the outcome of such a task, important changes in brain functioning may occur in the prefrontal cortex (responsible for executive functioning) at different levels (Ai et al., 2021), triggering changes in the individual’s psychological state, such as stress (Habay et al., 2021). However, there is a relationship between cognitive functioning, psychological state and HRV (Lin et al., 2017), as brain and heart connection is constant and it becomes important that these associated mechanisms act and react efficiently and modulated (You et al., 2021). However, the level of stress affects HRV *via* vagus nerve (parasympathetic) in the sinoatrial node of the heart, causing an imbalance between the sympathetic and parasympathetic systems (Quintana et al., 2012a; Kemp and Quintana, 2013). In addition, in athletes who are constantly performing tasks, whether in training or competitions, their performance can affect neurocardiac behavior and the results (win and defeat) will be critical to establishing balance (Britton et al., 2019; Broodryk et al., 2021). eSports players need of cognitive functioning at all times, especially concentration, in order to be efficient in the task (Machado et al., 2021) and this demand can provoke changes in the perceived stress and HRV, favoring or not game performance.

To the best of our knowledge, the study conducted by Broodryk et al. (2021) was the only one to investigate psychophysiological variables (i.e., cortisol, anxiety and mood) and their associations with victory and defeat in athletes. Therefore, future studies are needed to deepen the knowledge about HRV behavior and emotional states (not just stress) in different sports before and after different outcomes (win, lose and maybe draw), especially in eSports players. Studies with this line of investigation will also be relevant, with regard to the behavior of these variables in the competitive period, providing solid information for possible interventions in relation to the recovery process of athletes between games and throughout the competition.

This study results in important, albeit exploratory, findings for eSports athletes and coaches. In this sense, we can suggest that victory and defeat in competitive games elicit different responses in HRV and perceived stress. An important practical implication of our study deals with the use of HRV as an optimizing tool to the athletic training through the quantification of training loads (Borresen and Lambert, 2009), as well as the analysis of the training program (Tønnessen et al., 2014), allowing for an assessment, monitoring, recovery and improvement in the athletic performance. HRV is essential in monitoring fatigue and/or performance responses to overtraining or functional overload (Meeusen et al., 2013), which influences psychological factors (Oliveira-Silva et al., 2018; Mamlouk et al., 2021). In this sense, the most useful indicator of HRV is the rMSSD at rest (Buchheit, 2014). rMSSD is effective in identifying the general level of fatigue, although it does not allow the grouping of different sub-categories of fatigue (Buchheit, 2014). Thus, these findings should be taken into account by eSports athletes and coaches, as they can be used to assess, monitor, recovery and preserve efficiency in training and game performance.

Our study has some limitations. We investigated psychophysiological patterns of eSport athletes just before and after games and not during the game. In addition, we did not compare psychophysiological variables among athletes with different functions in the team. Another point is the lack of an assessment of other clinical variables related to HRV responses (e.g., anxiety, emotion-regulation) to control for. As future perspectives, topics such as the use of ergogenic resources to improve neurocognitive and neuromotor performance through caffeine (Sainz et al., 2020) and brain neuromodulation techniques, such as tDCS (Machado et al., 2021), should be explored.

## Conclusion

The present study investigated the behavior of HRV in the time and frequency domains, and in the perceived stress in different outcomes in eSports athletes during competitive matches. It was observed that VG had better HRV responses (greater parasympathetic activation) as well as lower levels of perceived stress, while DG had worst HRV responses (greater sympathetic activation) and higher levels of perceived stress. Future studies are needed to investigate HRV behavior and emotional states in eSports before and after different outcomes (win, lose and maybe draw).

## Data availability statement

The datasets presented in this article are not readily available because the data are under confidentiality requirements. Requests to access the datasets should be directed to SM, secm80@gmail.com.

## Ethical statement

The studies involving human participants were reviewed and approved by Ethics Committee of the Research Center in Sport, Health and Human Development (CIDESD; Portugal). The patients/participants provided their written informed consent to participate in this study.

## Author contributions

SM, BT, and DM have substantial contributions to the conception or design of the work, drafted the work and revising it critically for important intellectual content provide approval for publication of the content. LO, LC, DT, and FR helped in the acquisition, analysis, and interpretation of data for the work. All authors contributed to the article and approved the submitted version.

## Funding

This research was supported by national funds through the Portuguese Foundation for Science and Technology, I.P., under the project UID04045/2020.

## Conflict of interest

The authors declare that the research was conducted in the absence of any commercial or financial relationships that could be construed as a potential conflict of interest.

## Publisher’s note

All claims expressed in this article are solely those of the authors and do not necessarily represent those of their affiliated organizations, or those of the publisher, the editors and the reviewers. Any product that may be evaluated in this article, or claim that may be made by its manufacturer, is not guaranteed or endorsed by the publisher.
