# Corrigendum: Impact of victory and defeat on the perceived stress and autonomic regulation of professional eSports athletes

**DOI:** 10.3389/fpsyg.2022.1065664

**Published:** 2022-10-26

**Authors:** Sergio Machado, Leandro de Oliveira Sant'Ana, Luis Cid, Diogo Teixeira, Filipe Rodrigues, Bruno Travassos, Diogo Monteiro

**Affiliations:** ^1^Department of Sports Methods and Techniques, Federal University of Santa Maria, Santa Maria, Brazil; ^2^Department of Sports Science, University of Beira Interior, Covilhã, Portugal; ^3^Laboratory of Physical Activity Neuroscience, Neurodiversity Institute, Queimados, Brazil; ^4^Postgraduate Program in Physical Education, Federal University of Juiz de Fora, Juiz de Fora, Brazil; ^5^Research Center in Sport, Health and Human Development (CIDESD), Vila Real, Portugal; ^6^Sport Sciences School of Rio Maior, Polytechnic of Santarém (ESDRM-IPSantarém), Rio Maior, Portugal; ^7^Life Quality Research Centre (CIEQV), Leiria, Portugal; ^8^Faculty of Physical Education and Sport, Lusófona University, Lisbon, Portugal; ^9^Research Center in Sport, Physical Education, and Exercise and Health (CIDEFES), Lisbon, Portugal; ^10^ESECS, Polytechnic of Leiria, Leiria, Portugal; ^11^Portugal Football School, Portuguese Football Federation, Cruz Quebrada, Portugal

**Keywords:** victory, defeat, eSports, perceived stress, heart rate variability, HRV

In the published article, there were errors when citing some effect size and confidence interval values in the **Results** section.

A correction has been made to **Results**, “PSS-10,” paragraph 1. This sentence previously stated:

“PSS-10 in the post-game time was lower in VG than in DG (*p* ≤ 0.001; *d* = −16.62, CI 95%: −19.92 to −13.31, Figure 2).”

The corrected sentence appears below:

“PSS-10 in the post-game time was lower in VG than in DG (*p* ≤ 0.001; *d* = 16.92, CI 95%: 13.34 to 19.96, Figure 2).”

A correction has been made to **Results**, “PSS-10,” paragraph 2. This sentence previously stated:

“The interaction revealed a decreased score for PSS-10 in the post-game (4.33 ± 0.96) compared to BL (15.12 ± 1.77) and pre-game (14.75 ± 1.62) times for VG (*p* ≤ 0.001; *d* = −7.90, CI 95%: −10.13 to −5.07 and *d* =-7.44, CI 95%: −9.54 to −4.77 respectively), while also showed an increased score for PSS-10 in the post-game (27.79 ± 1.71) compared to BL (14.70 ± 1.60) and pre-game (14.20 ± 1.84) times for DG (*p* ≤ 0.001; *d* = −7.66, CI 95%: 5.50–9.23 and *d* = 7.91, CI 95%: 5.68–9.53, respectively).”

The corrected sentence appears below:

“The interaction revealed a decreased score for PSS-10 in the post-game (4.33 ± 0.96) compared to BL (15.12 ± 1.77) and pre-game (14.75 ± 1.62) times for VG (*p* ≤ 0.001; *d* = 7.58, CI 95%: 5.90 to 9.02 and *d* = 7.83, CI 95%: 6.09 to 9.31 respectively), while also showed an increased score for PSS-10 in the post-game (27.79 ± 1.71) compared to BL (14.70 ± 1.60) and pre-game (14.20 ± 1.84) times for DG (*p* ≤ 0.001; *d* = 7.91, CI 95%: 6.16 to 9.40 and *d* = 7.65, CI 95%: 5.95 to 9.11, respectively).”

A correction has been made to **Results**, “Heart rate variability,” paragraph 1. This sentence previously stated:

“In opposition, R-R in the post-game time was higher in VG than in DG (*p* ≤ 0.001; *d* = −12.81, CI 95%: −15.38 to −10.24, Figure 3A).”

The corrected sentence appears below:

“In opposition, R-R in the post-game time was higher in VG than in DG (*p* ≤ 0.001; *d* = 15.02, CI 95%: 11.83 to 17.73, Figure 3A).”

A correction has been made to **Results**, “Heart rate variability,” paragraph 1. This sentence previously stated:

“The interaction revealed an increased score of R-R in the post-game (993.44 ± 4.63) compared to BL (950.4 ± 39.97) and pre-game (951.28 ± 36.15) times for VG (*p* ≤ 0.0001; *d* = −0.76, CI 95%: −1.21 to −0.25 and *p* = 0.0002; *d* = −0.87, CI 95%: −1.34 to −0.34 respectively), while also showed a decrease in the score of R-R in the post-game (749.96 ± 22.46) compared to BL (948.36 ± 37.02) and pre-game (949.52 ± 33.56) times for DG (*p* ≤ 0.001; *d* =-6.67, CI 95%: −8.56 to −4.27 and *d* = −6.39, CI 95%: −8.21 to −4.08, respectively).”

The corrected sentence appears below:

“The interaction revealed an increased score of R-R in the post-game (993.44 ± 4.63) compared to BL (950.4 ± 39.97) and pre-game (951.28 ± 36.15) times for VG (*p* ≤ 0.0001; *d* = 1.51, CI 95%: 0.86 to 2.12 and *p* = 0.0002; *d* = 1.64, CI 95%: 0.97 to 2.25 respectively), while also showed a decrease in the score of R-R in the post-game (749.96 ± 22.46) compared to BL (948.36 ± 37.02) and pre-game (949.52 ± 33.56) times for DG (*p* ≤ 0.001; *d* = 6.48, CI 95%: 5.01 to 7.75 and *d* = 6.99, CI 95%: 5.42 to 8.34, respectively).”

A correction has been made to **Results**, “Heart rate variability,” paragraph 2. This sentence previously stated:

“However, SDNN in the post-game time was higher in VG than in DG (*p* ≤ 0.001; *d* = 3.73, CI 95%: 2.81–4.65, Figure 3B).”

The corrected sentence appears below:

“However, SDNN in the post-game time was higher in VG than in DG (*p* ≤ 0.001; *d* = 3.73, CI 95%: 2.76 to 4.58, Figure 3B).”

A correction has been made to **Results**, “Heart rate variability,” paragraph 2. This sentence previously stated:

“The interaction revealed an increased score of SDNN in the post-game (70.76 ± 3.75) compared to BL (61.6 ± 4.91) and pre-game (61.76 ± 4.9) times for VG (*p* ≤ 0.001; *d* = 2.12, CI 95%: 1.41–2.67 and *d* = 2.08, CI 95%: 1.38–2.63, respectively), while also showed a decreased score of SDNN in the post-game (57.48 ± 3.36) compared to BL (61.92 ± 4.97) and pre-game (62.28 ± 4.41) times for DG (*p* =0.001; *d* =-1.07, CI 95%: −1.56 to −0.48 and *p* ≤ 0.001; *d* = −1.24, CI 95%: −1.76 to −0.61, respectively).”

The corrected sentence appears below:

“The interaction revealed an increased score of SDNN in the post-game (70.76 ± 3.75) compared to BL (61.6 ± 4.91) and pre-game (61.76 ± 4.9) times for VG (*p* ≤ 0.001; *d* = 2.13, CI 95%: 1.41 to 2.79 and *d* = 2.06, CI 95%: 1.35 to 2.71, respectively), while also showed a decreased score of SDNN in the post-game (57.48 ± 3.36) compared to BL (61.92 ± 4.97) and pre-game (62.28 ± 4.41) times for DG (*p* = 0.001; *d* = 1.05, CI 95%: 0.44 to 1.62 and *p* ≤ 0.001; *d* = 1.22, CI 95%: 0.61 to 1.81, respectively).”

A correction has been made to **Results**, “Heart rate variability,” paragraph 3. This sentence previously stated:

“However, rMSSD in the post-game time was higher in VG than in DG (*p* ≤ 0.001; *d* = 1.67, CI 95%: 1.02–2.31, Figure 3C).”

The corrected sentence appears below:

“However, rMSSD in the post-game time was higher in VG than in DG (*p* ≤ 0.001; *d* = 2.79, CI 95%: 1.97 to 3.52, Figure 3C).”

A correction has been made to **Results**, “Heart rate variability,” paragraph 3. This sentence previously stated:

“The interaction revealed an increased score of rMSSD in the post-game (76.96 ± 2.31) compared to BL (72.96 ± 3.80) and pre-game (72.12 ± 2.18) times for VG (*p* ≤ 0.001; *d* = 1.31, CI 95%: 0.78–1.76 and *p* = 0.003; *d* = 2.16, CI 95%: 1.44–2.72, respectively), while also showed a decreased score of rMSSD in the post-game (64.4 ± 5.93) compared to BL (73.24 ± 3.46) and pre-game (72.8 ± 3.42) times for DG (*p* ≤ 0.001; *d* =-1.88, CI 95%: −2.54 to −1.08 and *d* = −1.80, CI 95%: −2.43 to −1.02, respectively).”

The corrected sentence appears below:

“The interaction revealed an increased score of rMSSD in the post-game (76.96 ± 2.31) compared to BL (72.96 ± 3.80) and pre-game (72.12 ± 2.18) times for VG (*p* ≤ 0.001; *d* = 1.27, CI 95%: 0.65 to 1.86 and *p* = 0.003; *d* = 2.15, CI 95%: 1.43 to 2.81, respectively), while also showed a decreased score of rMSSD in the post-game (64.4 ± 5.93) compared to BL (73.24 ± 3.46) and pre-game (72.8 ± 3.42) times for DG (*p* ≤ 0.001; *d* = 1.82, CI 95%: 1.14 to 2.45 and *d* = 1.74, CI 95%: 1.06 to 2.36, respectively).”

A correction has been made to **Results**, “Heart rate variability,” paragraph 4. This sentence previously stated:

“In opposition, pNN50 in the post-game time was higher in VG than in DG (*p* ≤ 0.001; *d* = 2.86, CI 95%: 2.07–3.65, Figure 3D).”

The corrected sentence appears below:

“In opposition, pNN50 in the post-game time was higher in VG than in DG (*p* ≤ 0.001; *d* = 2.85, CI 95%: 2.03 to 3.95, Figure 3D).”

A correction has been made to **Results**, “Heart rate variability,” paragraph 4. This sentence previously stated:

“The interaction revealed an increased score of pNN50 in the post-game (7.04 ± 1.45) compared to BL (3.28 ± 1.13) and pre-game (3.48 ± 0.87) times for VG (*p* ≤ 0.001; *d* = 2.91, CI 95%: 2.02 to 3.60 and *d* = 3.07, CI 95%: 2.13–3.78 respectively), while also showed no significant changes in the score of pNN50 in the post-game (3.64 ± 0.86) compared to BL (3.38 ± 1.18) and pre-game (3.92 ± 1.11) times for DG (*p* = 0.988 and *p* = 0.999, respectively).”

The corrected sentence appears below:

“The interaction revealed an increased score of pNN50 in the post-game (7.04 ± 1.45) compared to BL (3.28 ± 1.13) and pre-game (3.48 ± 0.87) times for VG (*p* ≤ 0.001; *d* = 2.89, CI 95%: 2.06 to 3.83 and *d* = 2.98, CI 95%: 2.13 to 3.73 respectively), while also showed no significant changes in the score of pNN50 in the post-game (3.64 ± 0.86) compared to BL (3.38 ± 1.18) and pre-game (3.92 ± 1.11) times for DG (*p* = 0.988 and *p* = 0.999, respectively).”

A correction has been made to **Results**, “Heart rate variability,” paragraph 5. This sentence previously stated:

“Considering frequency domain measures, for HF any difference was found between groups in the BL (*p* = 0.993) and pre-game (*p* = 0.999) times, nor intra group difference between BL and pre-game times for VG (*p* = 0.999) and for DG (*p* = 0.999). HF in the post-game times was higher in VG than in DG (*p* ≤ 0.0001; *d* = 3.35, CI 95%: 2.49–4.21, Figure 4A).”

The corrected sentence appears below:

“Considering frequency domain measures, for HF any difference was found between groups in the BL (*p* = 0.993) and pre-game (*p* = 0.999) times, nor intra group difference between BL and pre-game times for VG (*p* = 0.999) and for DG (*p* = 0.999). HF in the post-game times was higher in VG than in DG (*p* ≤ 0.0001; *d* = 5.09, CI 95%: 3.89 to 6.14, Figure 4A).”

A correction has been made to **Results**, “Heart rate variability,” paragraph 5. This sentence previously stated:

“The interaction revealed an increased score of HF in the post-game (8.28 ± 1.20) compared to BL (6.08 ± 1.57) and pre-game (5.96 ± 0.88) times for VG (*p* ≤ 0.001; *d* = 1.59, CI 95%: 1.00–2.07 and *d* = 2.23, CI 95%: 1.50–2.80, respectively), however with no significant differences between times for while also showed a decreased score of HF in the post-game (3.24 ± 0.72) compared to BL (6.04 ± 1.54) and pre-game (5.88 ± 0.97) times for DG (*p* ≤ 0.001; *d* = −2.48, CI 95%: −3.27 to −1.49 and *d* = −3.12, CI 95%: −4.07 to −1.93, respectively).”

The corrected sentence appears below:

“The interaction revealed an increased score of HF in the post-game (8.28 ± 1.20) compared to BL (6.08 ± 1.57) and pre-game (5.96 ± 0.88) times for VG (*p* ≤ 0.001; *d* = 1.57, CI 95%: 0.92 to 2.18 and *d* = 2.20, CI 95%: 1.47 to 2.87, respectively), however with no significant differences between times, while also showed a decreased score of HF in the post-game (3.24 ± 0.72) compared to BL (6.04 ± 1.54) and pre-game (5.88 ± 0.97) times for DG (*p* ≤ 0.001; *d* = 2.33, CI 95%: 1.58 to 3.01 and *d* = 3.09, CI 95%: 2.23 to 3.86, respectively).”

A correction has been made to **Results**, “Heart rate variability,” paragraph 6. This sentence previously stated:

“However, LF in the post-game time was lower in VG than in DG (*p* ≤ 0.0001; *d* = −2.87, CI 95%: −3.66 to −2.08, Figure 4B).”

The corrected sentence appears below:

“However, LF in the post-game time was lower in VG than in DG (*p* ≤ 0.0001; *d* = 4.15, CI 95%: 3.13 to 5.09, Figure 4B).”

A correction has been made to **Results**, “Heart rate variability,” paragraph 6. This sentence previously stated:

“The interaction revealed a decreased score of LF in the post-game (5.16 ± 0.85) compared to BL (7.72 ± 1.02) and pre-game (8.08 ± 0.86) times for VG (*p* ≤ 0.001; *d* = −2.74, CI 95%: −3.59 to −1.67 and *d* = −3.42, CI 95%: −4.44 to −2.12, respectively), while also showed an increased score of LF in the post-game (9.24 ± 1.09) compared to BL (7.8 ± 0.81) and pre-game (7.96 ± 0.88) times for DG (*p* ≤ 0.001; *d* = 1.52, CI 95%: 0.94–1.99 and *d* = 1.30, CI 95%: 0.77–1.75, respectively).”

The corrected sentence appears below:

“The interaction revealed a decreased score of LF in the post-game (5.16 ± 0.85) compared to BL (7.72 ± 1.02) and pre-game (8.08 ± 0.86) times for VG (*p* ≤ 0.001; *d* = 2.73, CI 95%: 1.92 to 3.45 and *d* = 3.42, CI 95%: 2.50 to 4.22, respectively), while also showed an increased score of LF in the post-game (9.24 ± 1.09) compared to BL (7.8 ± 0.81) and pre-game (7.96 ± 0.88) times for DG (*p* ≤ 0.001; *d* = 1.50, CI 95%: 0.85 to 2.10 and *d* = 1.29, CI 95%: 0.66 to 1.88, respectively).”

A correction has been made to **Results**, “Heart rate variability,” paragraph 7. This sentence previously stated:

“In opposition, LF-HF in the post-game time was lower in VG than in DG (*p* ≤ 0.001; *d* = −2.60, CI 95%: −3.35 to −1.84, Figure 4C).”

The corrected sentence appears below:

“In opposition, LF-HF in the post-game time was lower in VG than in DG (*p* ≤ 0.001; *d* = 2.59, CI 95%: 1.80 to 3.30, Figure 4C).”

A correction has been made to **Results**, “Heart rate variability,” paragraph 7. This sentence previously stated:

“The interaction revealed a decreased score of LF-HF in the post-game (1.42 ± 0.20) compared to BL (2.52 ± 0.78) and pre-game (2.44 ± 0.70) times for VG (*p* ≤ 0.001; *d* = −2.24, CI 95%: −2.98 to −1.33 and *d* = −2.27, CI 95%: −3.01 to −1.35, respectively), while also showed an increased score of LF-HF in the post-game (3.18 ± 0.94) compared to BL (2.41 ± 0.65) and pre-game (2.34 ± 0.66) times for DG (*p* = 0.004; *d* = 0.97, CI 95%: 0.49–1.38 and *p* = 0.002; *d* = 1.05, CI 95%: 0.56–1.47, respectively).”

The corrected sentence appears below:

“The interaction revealed a decreased score of LF-HF in the post-game (1.42 ± 0.20) compared to BL (2.52 ± 0.78) and pre-game (2.44 ± 0.70) times for VG (*p* ≤ 0.001; *d* = 1.93, CI 95%: 1.23 to 2.57 and *d* = 1.98, CI 95%: 1.28 to 2.62, respectively), while also showed an increased score of LF-HF in the post-game (3.18 ± 0.94) compared to BL (2.41 ± 0.65) and pre-game (2.34 ± 0.66) times for DG (*p* = 0.004; *d* = 0.95, CI 95%: 0.35 to 1.52 and *p* = 0.002; *d* = 1.03, CI 95%: 0.43 to 1.61, respectively).”

The authors apologize for these errors and state that they do not change the scientific conclusions of the article in any way. The original article has been updated.

## Publisher's note

All claims expressed in this article are solely those of the authors and do not necessarily represent those of their affiliated organizations, or those of the publisher, the editors and the reviewers. Any product that may be evaluated in this article, or claim that may be made by its manufacturer, is not guaranteed or endorsed by the publisher.

